# Malignant catarrhal fever in pastoral Maasai herds caused by wildebeest associated alcelaphine herpesvirus-1: An outbreak report

**Published:** 2013

**Authors:** Emanuel Senyael Swai, Angolwise Mwakibete Kapaga, Francis Sudi, Potari Meshack Loomu, Gladyness Joshua

**Affiliations:** 1 *Veterinary Investigation Centre (VIC), Arusha, Tanzania;*; 2 *Central Veterinary Laboratory (CVL), Dar-es-Salaam, Tanzania; *; 3 *Epidemiology Unit, Ministry of Livestock and Fisheries Development, Dar-es-Salaam, Tanzania; *; 4 *District Veterinary Office, Loliondo, Arusha, Tanzania.*

**Keywords:** Bovine, MCF, Tanzania, Wildebeest, Wildlife-livestock ecosystem

## Abstract

An outbreak of malignant catarrhal fever (MCF), a fatal viral disease in indigenous Tanzanian shorthorn zebu in Ngorongoro district of Tanzania during the period of June 2004 has been described. The disease was diagnosed by clinical, post mortem findings and the virus was identified using molecular characterization study. The history and clinical features included pyrexia, cornel opacity, nasal discharges, multifocal buccal ulceration of varying size and general unthrifty. Pathological features showed that abomasum and intestine contents were blood tinged and their walls were congested and hyperemic with scattered hemorrhagic patches. Furthermore greenish-black longitudinal stripes in the caecum and ileo-caecal junction that disappeared upon opening of the intestine were a distinct feature. It has been concluded that as the wildebeest have a wide distribution throughout Tanzania, it is likely that MCF occurs in many parts of the country and therefore continuation of surveillance system seems necessary.

## Introduction

Malignant catarrhal fever (MCF) is a dramatic, fatal, systemic lymphoproliferative disease of cattle and a variety of other susceptible ungulates.^1^^,^^2^ The disease manifests itself as an acute, non-contagious disease of cattle, with low morbidity (estimated at 1-3%) and high mortality (90-100%). The known causative agents are two viruses of the gamma herpesvirus subfamily (*Rhadinovirus* genus) - alcelaphine herpesvirus-1 (AlHV-1) and ovine herpesvirus-2 (OvHV-2) that cause no apparent disease in their natural reservoir hosts but severe disease in susceptible species that are in proximity to the natural hosts. The natural reservoir host for AlHV-1 is the wildebeest (*Connochaetes* *spp.*) and this virus is therefore restricted to sub-Saharan Africa^3^ and occasionally in zoological collections elsewhere. Thus, AlHV-1 MCF in Africa is a consequence of cattle being in the vicinity of wildebeest. Natural MCF-susceptible species include cattle,^3^ farmed deer,^4^ bison,^5^ buffalo (*Bubalus bubalis)* and Bali cattle^6^ and (non-ruminant) pigs.^7^ Rabbits, hamsters can be experimentally infected with MCF virus. The mechanism whereby the viruses cause this immune dysregulation in susceptible animals and not in the reservoir hosts is unknown.^2^

In terms of welfare importance and economic impact, the predominant form in Africa is AlHV-1 MCF, whereas in the rest of the world it is OvHV-2 MCF. In terms of wealth and quality of life, the disease can have profound consequences for pastoralists and farmers in parts of the developing world where their livestock are in contact with the reservoir species such as southern and eastern Africa. The impact is particularly severe for the Maasai in Africa.^8^^,^^9^ The present report is based on an outbreak of wildebeest associated malignant catarrhal fever in an area of wild life-livestock interface ecosystem in Ngorongoro (northern Tanzania).

## Materials and Methods

**Outbreak area.** Ngorongoro is one of the six districts of Arusha region bordering Kenya to the north and the Serengeti National Park to the west. It lies between Latitude 2 to 4 South and Longitude 35 and 36 East and is an extensive sparsely populated rangeland covering 15,431 km^2^ and an integral part of the Serengeti and the pastoral ecosystems of both Tanzania and Kenya. The livestock population is estimated at 300,000 heads of cattle and 450,000 small ruminants. The livestock interact with almost two million of various species of wildlife as they share grazing, water and salt-licking points especially during the wet season.^9^ The interactions with wildlife combined with the semi-nomadic mode of livestock production result in a unique and complex disease situation in this area.

**Case history. **The disease was first reported to the nearest District Veterinary Office on 16^th^ April 2004. The affected village, Engusero sambu is at the Kenya border. The information showed that a number of cattle (n = 12) had died from a disease, which apparently did not respond to antibiotic treatment. Grown up calves (1 to 2 years old) were noted to be affected more than other age groups. A tentative diagnosis of MCF was made and the area and other competent authorities (Director of Veterinary Service) were informed as per the regulations. Differential diagnoses were: MCF, rinderpest, bovine viral diarrhea/mucosal disease (BVD/MD), foot and mouth disease (FMD) and infectious bovine rhinotracheitis (IBR).

**Clinical findings. **The clinical episodes observed in all identified sick animals were generally similar. The animal had anorexia, slightly laboured breathing, diarrhoeic, rectal temperature between 38.5 to 41.5 ˚C, purulent nasal discharges, dental pad necrotic erosion, lacrimation, bilateral cornea opacity, loss of body condition, a typical stomatitis-enteritis syndrome.^10^ Other signs included nervous symptoms syndrome i.e. muscle tremor around the shoulder and hump. 

**Gross pathological lesions. **From the two autopsied animals, the following were the major lesions encountered. Upon opening of the abdominal cavity, 150-200 mL of straw colored fluid was consistently evident. The contents of the abomasum and small/large intestine were blood tinged and the wall was congested and hyperaemic with scattered haemorrhagic patches. Greenish-black longitudinal stripes in the caecum and ileo-caecal junction that disappeared upon opening of the intestine were a distinct feature. The liver looked mottled in appearance and enlarged with bulged edges. The lungs were emphysematous, pale reddish and light. Blood stained fluid oozed out from cut surfaces of the lungs. The trachea and bronchi were filled with mucopurulent exudates. The pericardial sac was filled with excessive amount of straw colored fluid. In some of the cases ecchymotic haemorrhages were also observed in the epicardium and the pericardium. No gross lesions were seen in the brain but it appeared wet and a bit enlarged with a black stain on the front lobes.

**Laboratory and field test results. **Collected sera (39) and eye swabs (37) from the animals which were manifesting stomatitis-enteritis syndrome were subjected to rinderpest pen side test (clearview rinderpest test- for rinderpest antigen detection) and competitive ELISA* (C*-ELISA*) *for rinderpest antibodies detection for possible rinderpest rule out. All samples were negative. Proceeding of the samples (brain, spleen, lung, liver) were referred to *Office International des Epizooties* (OIE), Regional Reference laboratory, Ondestepoort Veterinary institute (ovi, South Africa) for further laboratory procedure and testing for malignant catarrhal fever (MCF).

**Genome characterisation. **Tissue samples (brain and spleen) forwarded to OVI revealed a positive diagnosis for AlHV-1 associated MCF. Furthermore, AlHV-1, DNA genome was amplified using nested polymerase chain reaction (PCR) and sequenced.^11^^,^^12^ The sequence analysis data on the AlHV-1, DNA genome proved to be wildebeest associated malignant catarrhal fever.

## Discussion

The clinical signs of MCF described in the present outbreak, which are similar to descriptions from other countries, are indistinguishable from those of rinderpest through visual and physical examination.^13^^,^^14^ Although clinically indistinguishable from rinderpest, the cause is a different virus which is also distinct from the viruses of foot and mouth disease (FMD), bovine viral diarrhea (BVD) that express less similar clinical manifestations.^15^^,^^16^ In the recent past (over the last 20 years) there has been a confirmed outbreak of MCF in Tanzania.^13^^,^^17^ A participatory disease prioritization study conducted in Maasai rangeland, northern Tanzania, identified and ranked MCF as the disease of most concern, above East Coast Fever (ECF) and contagious bovine pleural pneumonia (CBPP).^13^ Further more results of genome characterisation study revealed AlHV-1 virus which was associated with wildebeest. Disease transmission in wildebeest calves occurred during calving period. Calves become infected within the first few months of age and do excrete high levels of cell free virus in ocular–nasal secretion during the 3-4 months of life.^18^^,^^19^ It is likely therefore that transmission from wildebeest to cattle occur through aerosol, ingestion of contaminated pasture or water with afterbirths materials or ingestion of hair from wildebeest calves (coinciding with the time that calves moults at 3-4 month of age).^1^ Wildebeest have a wide distribution throughout Tanzania and it’s likely that MCF occurs in many parts of the country. However, cases are rarely reported to veterinary authorities and official incidence data are difficult to obtain and not known.^9^^,^^13^^,^^20^

In this outbreak, no attempt was made to undertake detailed investigation on the role of wildebeest and small ruminants (sheep, goats, wild ungulates) in the epidemiology of the disease. Resources constraints affecting logistics and laboratory capacity were the main reason. These animal species are known to be susceptible reservoirs and carriers of MCF virus.^1^^,^^2^ However, the outbreak of MCF described in the present report could have been associated with the close interaction with the wildebeest, which are on move between northern Tanzania and Kenya border during their migratory periods. It is recommended that, since contact between wildebeest cannot be excluded in the livestock-wildlife interface areas of northern Tanzania, continuation of surveillance system should be in place. Additionally, formal structured study, which will characterize disease incidence, losses and mortality, is proposed.

## References

[1] Barnard BJ, Van de Pypekamp HE, Griessel MD (1989). Epizootology of wildebeest-derived malignant catarrhal fever in an outbreak in the north-western Transvaal: Indications of an intermediate host. Onderstepoort J Vet Res.

[2] Hunt RD, Billups LH (1979). Wildebeest-associated malignant catarrhal fever in Africa: A neoplastic disease of cattle caused by an oncogenic herpesvirus. Comp Immunol Microbiol Infect Dis.

[3] Plowright W (1968). Malignant catarrhal fever. J Am Vet Med Assoc.

[4] Reid RW, Buxton D, Corrigal W (1979). An outbreak of malignant catarrhal fever in red deer (Cervus elephus). Vet Rec.

[5] O’ Toole D, Lih H, Sourk C (2002). Malignant catarrhal fever in a bison (bison bison) feedlot, 1993-2000. J Vet Diagn Invest.

[6] Ramachandran S, Malole M, Rifuliadi D (1982). Experimental reproduction of malignant catarrhal fever in Bali cattle (Bos sondaicus). Aust Vet J.

[7] Loken T, Aleksandersen M, Reid H (1998). Malignant catarrhal fever caused by ovine herpesvirus-2 in pigs in Norway. Vet Rec.

[8] Mushi EZ, Rurangirwa FR (1982). Epidemiology of bovine malignant catarrhal fever, a review. Vet Res Commun.

[9] McCabe JT, Schofield EC, Nygaard Pederson G, Thompson DM (1997). Multiple land-use: The experience of the Ngorongoro conservation area.

[10] Obi TU, Roeder PL, Geering WA (1986). FAO-PARC, Recognizing rinderpest, field manual.

[11] Vilcek S, Nettleton P, Herring JA (1994). Rapid detection of bovine herpes virus 1 (BHV 1) using the polymerase chain reaction. Vet Microbiol.

[12] Traul DL, Elias S, Taus NS (2005). A real-time PCR assay for measuring alcelaphine herpesvirus-1 DNA. J Virol Methods.

[13] Kalunda M, Shaka S, Henrikson K (1982). An epizootic of bovine malignant catarrhal fever in northern Tanzania in 1976. Bull Anim Hlth and Prod Afri.

[14] Ngotho RN, Mirangi PK, Mimano LN (1999). Costs of malignant catarrhal fever (MCF) in group and private ranches in Kajiado and Narok Districts of Kenya.

[15] Msolla P, Sinclair JA, Nettleton P (1988). Prevalence of antibodies to bovine virus diarrhea-mucosal disease virus in Tanzanian cattle. Trop Anim Health Prod.

[16] Hyera JM, Liess B, Frey HR (1991). Bovine viral diarrhea virus infection in cattle, sheep and goats in northern Tanzania. Trop Anim Health Prod.

[17] Cleaveland S, Kusiluka L, ole Kuwai J (2001). Assessing the impact of malignant catarrhal fever in Ngorongoro District.

[18] Plowright W (1986). Malignant catarrhal fever. Rev Sci Tech Off Int Epiz.

[19] Mushi EZ, Karstad L, Jessett DM (1980). Isolation of bovine malignant catarrhal fever virus from ocular and nasal secretions of wildebeest calves. Res Vet Sci.

[20] Bourn D, Blench R (1999). Can livestock and wild life co-exist? An interdisciplinary approach.

